# Neonatal Sacrococcygeal Teratoma: Our Experience with 10 Cases

**Published:** 2013-01-01

**Authors:** Shalini Sinha, Yogesh Kumar Sarin, Vidyanand P Deshpande

**Affiliations:** Department of Pediatric Surgery, Maulana Azad Medical College, New Delhi-1100021

**Keywords:** Sacrococcygeal teratoma

## Abstract

Aim: To analyse the outcome of neonatal sacrococcygeal teratomas (SCT) in our setup.

Materials and Methods: Hospital records of 10 neonates, who were operated for SCT during 14 years time period, were retrieved and analysed. Letters were sent to 6 parents/ caretakers of children who were lost to follow up; none of them responded.

Results: Seven girls and 3 boys with a mean age of 9 days (range 1-30 days) underwent excision of SCT in the neonatal period. Antenatal pickup rates were poor (2/10). Two patients presented with tumor rupture. Though all had an obvious mass at birth, only half of them presented on day 1 of life. The remaining 5 patients came late at a mean age of 11 days. Half of the SCTs were 10 cm or larger in size. One patient was misdiagnosed as meningomyelocoele. All underwent complete excision with coccygectomy by posterior approach in prone position. There were only 2 patients who could be classified as Altman Type II, the rest were all Altman Type I. Histopathology (HPE) revealed mature cystic teratoma (n=8), grade 1 immature teratoma (n=1) and grade 3 immature teratoma (n=1). There was no mortality; and complications were seen in 3/10 patients (1 neurogenic bladder, 1 major wound infection with ventriculitis and 1 minor wound infection). The mean follow up was 25 months (range 1 month to 6 years) in 4 patients with no recurrence.

Conclusions: Neonatal SCTs are usually benign with a good outcome after complete surgical excision with a low complication rate. Although long term follow up has been advocated, the follow up was poor in this series.

## INTRODUCTION

Sacrococcygeal teratoma (SCT) has an incidence of about 1/40,000 live births and is the commonest congenital tumour in the neonate [1]. In 1973, Altman classified SCT into 4 types based on the external component and intrapelvic/ intra-abdominal extension of the tumour (American Academy of Pediatrics Surgical Section classification) [1]. The SCTs seen at birth are usually Altman Type I and II (87%) [1]. Rarely Type III can also be seen in neonates [2]. Type IV is typically seen later in life as there is no external component [1]. We describe our experience with 10 neonatal SCTs and review the pertinent literature.

## MATERIALS AND METHODS

A retrospective descriptive study was carried out in one of the two units of Pediatric Surgery, in a tertiary care public hospital of a resource challenged country over fourteen years four months (May 1998 to August 2012). The hospital records of 10 neonates, who underwent surgical treatment for SCT during this time period, were retrieved and analysed. Letters were sent to the parents of 6 children, who were lost to follow up (LTFU), but none of them responded. We also attempted telephoning at their contact numbers obtained from hospital records. However not a single patient’s family could be reached. During the same period, a total of 32 patients of SCT of all age groups were treated in our Pediatric Surgical Unit.

## RESULTS

Seven girls and 3 boys with a mean age of 9 days (range 1-30 days) underwent excision of SCT in the neonatal period. Antenatal diagnosis of sacrococcygeal mass (SCM) was made only in 2/3 mothers who had 3rd trimester ultrasonograms. Polyhydromnios without placentomegaly was seen in 1 mother. None had family history of twinning. The mean birth weight was 2710g (range 1975 - 3500g) and all except 2 were full-term babies. Two neonates were born by lower segment Caesarean section (LSCS) and the rest were normal vaginal deliveries (NVD). Two out of eight NVDs were unsupervised home deliveries. 

All of them had an obvious mass at lower back since birth (Fig. 1). Despite the presence of an obvious mass, only half of them were brought to hospital on day 1 of life. A delay of 5-18 days (mean 11 days) was seen in the other half of the patients. One antenatally diagnosed baby born by LSCS had rupture of the tumor with bleeding and had to be rushed for surgery. Another baby born by NVD at home presented at 11 days with rupture but no bleeding and was mistaken for a meningomyelocoele (MMC) preoperatively as the child also had an equinus deformity of right foot. One term baby born elsewhere by LSCS for meconium stained liquor, was referred to us with SCT (missed on 3rd trimester scan) and severe respiratory distress since 2 hours of life. Chest X-Ray revealed bilateral ground glass appearance and there was no evidence of cardiac failure. Echocardiography was normal. The baby was stabilized by neonatologists for probable meconium aspiration syndrome. Surgery was deferred till day 30 of life when his respiratory conditions had completely settled so that general anaesthesia could be given safely. Associated anomalies included cleft lip and palate in 1 patient and equinus deformity of foot in another. In half of them, the tumor size was 10 cm or more, the largest being 20 cms. Alfa-feto protein (AFP) was not done. 

**Figure:**
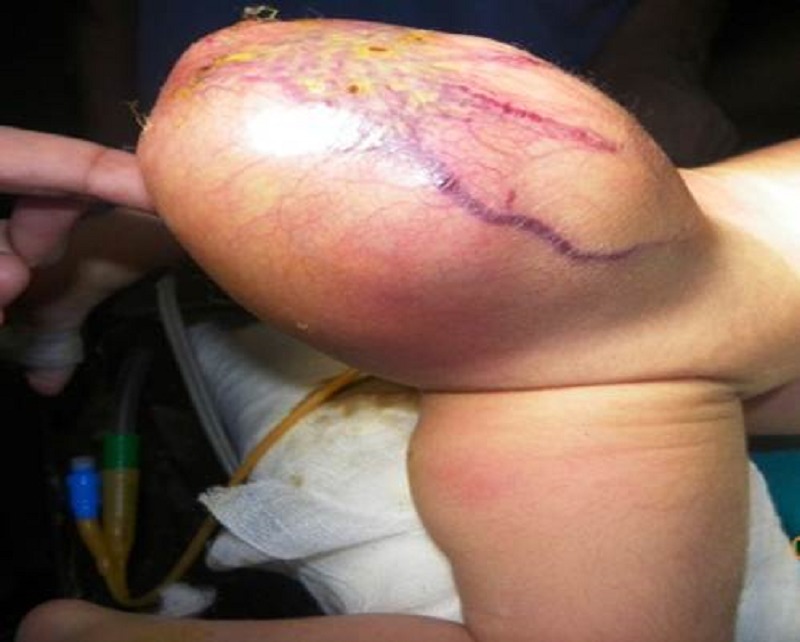
Figure 1: SCT

After preoperative stabilization, the neonates underwent complete excision of SCT with coccygectomy (Fig. 2) by posterior approach in prone position through an inverted chevron incision. Laparotomy was not required in any case. There were only 2 patients who could be classified as Altman Type II, the rest being Altman Type I. All neonates needed intra-operative blood transfusion. Postoperative ventilation was necessary in only 1 baby. All excised specimens were subjected to histopathological examination (HPE), which revealed mature teratoma (n=8), grade 1 immature teratoma (n=1) and grade 3 immature teratoma (n=1).

**Figure:**
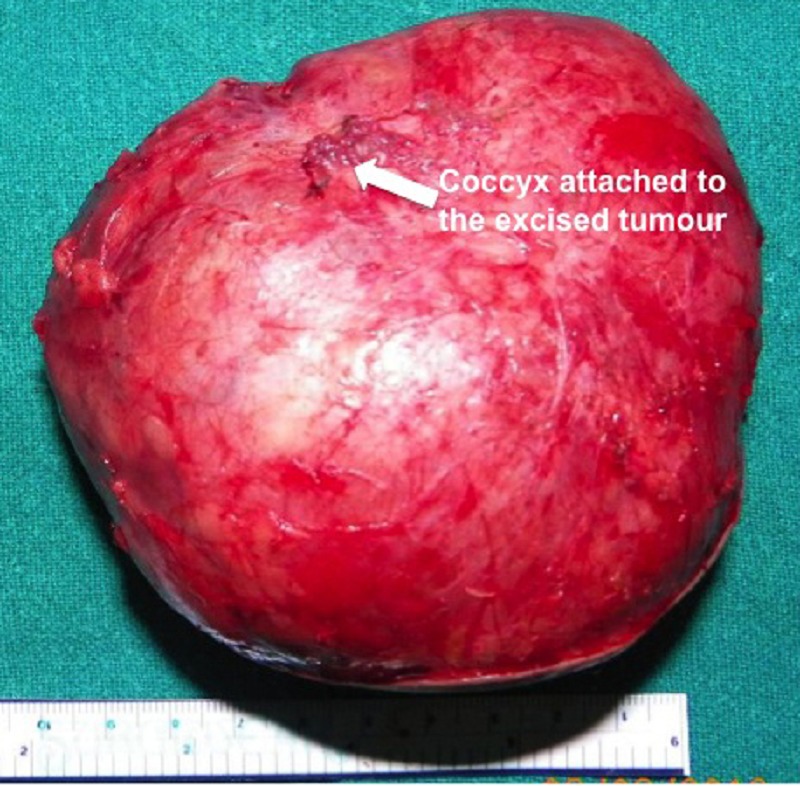
Figure 2: Excised specimen.

There were no deaths. Early postoperative complications included minor wound infection (n=1) and major wound infection with ventriculitis (n=1). Neurogenic bladder was seen in 1 patient who required vesicostomy 1 month after excision of SCT. She underwent vesicostomy closure at the age of 3 years and parents were advised clean intermittent catheterization (CIC). However, there was poor compliance to the suggested management and she developed recurrent urinary tract infections (UTI). At 6 years follow-up, her micturating cystourethrogram (MCUG) (Fig. 3) and radionucleiotide renal scan (DMSA) showed major vesicoureteric reflux (VUR) on left side and multiple scars in the left kidney. Increased frequency of stools was seen in 2 patients during the first few months after surgery.

**Figure:**
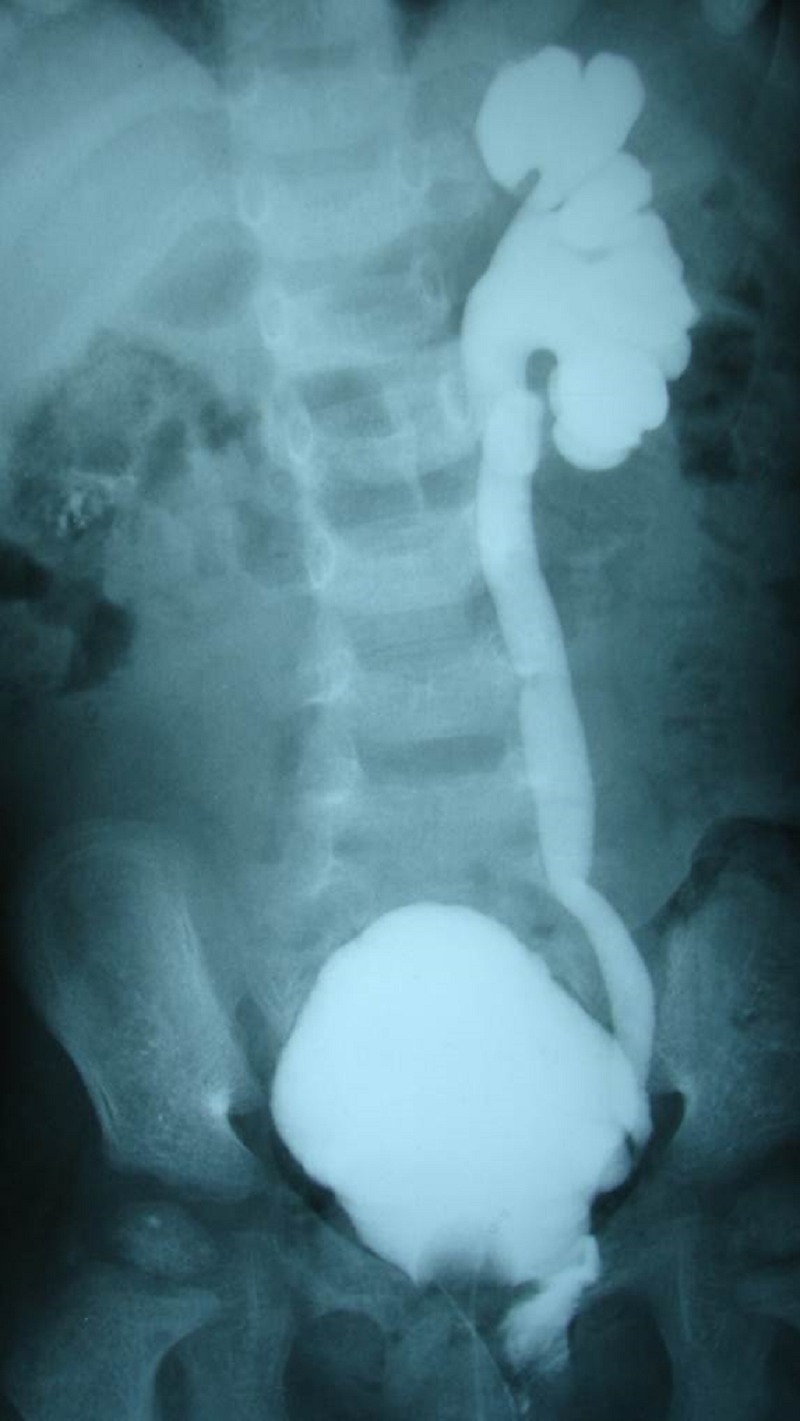
Figure 3: MCUG showing VUR.

All patients were advised long term follow up, however only 4 have come regularly. The mean follow-up was 25 months (range 1 m to 6 years) with no recurrence.

## DISCUSSION

Although there is no dearth of literature on SCT, there are very few case series from resource challenged nations like ours [3, 4]. 

Male:Female ratio: A female preponderance (2.3:1) was noted in our series consistent with the female: male ratio of 3-4: 1 in literature [1, 5, 6]. The exact reason for this predilection is not known. 

Size: Altman et al have classified the size of SCTs as follows: small, 2 to 5 cm diameter; moderate, 5 to 10 cm diameter; large, >10 cm diameter [1]. Going by this classification, half of our cases were large tumours measuring >10cm. Although some authors believe that the size of SCT is independent of its biological behaviour [6]; larger tumours are more likely to have immature histology and may lead to greater intraoperative blood loss [1, 7]. Both the tumours with immature histology in our study were larger than 10 cms in size.

Type: The most common type seen was Type I (8/10) followed by Type II (2/10). There were no Type III/IV SCTs as they are known to occur beyond neonatal age.

Differential diagnosis: Lemire et al have described 50 differential diagnoses for SCT [8]. However, type I /II SCT should mainly be differentiated from a sacral meningomyelocoele (MMC) which was challenging in one of our cases. An MRI could have helped in differentiating between the two entities.

Histology: Histological grading of SCTs (Gonzalez-Crussi) from grade 0-3 is based on the absence/ presence of immature neural elements and their quantities [9]. The incidence is reported as benign (grade 0) - 75%, immature (grades 1-3) - 11.8%, and malignant - 13.2% [10]. Some authors believe that this does not correlate with the prognosis of SCT [6]. As was seen in our patients, the incidence of immature teratomas is reported as about 20% [7, 11] and they are usually found in newborns [6]. 

Associated Malformations: Associated malformations are seen in 18% of SCTs which is comparable to 20% in this series (2/10) [1, 5]. The most commonly seen anomalies are anorectal and genital [12] which we did not see. Lahdenne et al have reported a high incidence of vertebral abnormalities (80%) in 45 patients with benign SCT at a mean follow up of 21 years [13]. The association of SCT with cleft lip and palate, as seen in one of our patients, could not be replicated in the literature search. This observation could be incidental as no possible embryological event can explain this concurrence.

Antenatal Diagnosis: Type I and II SCTs are commonly diagnosed by prenatal sonogram in the 24th – 34th weeks of gestation [12]. The presence of a heterogeneous, well circumscribed exophytic mass at the caudal end of the fetus is pathognomonic. Even large Type III and IV tumors can be diagnosed prenatally [14]. A close antenatal observation is necessary to look for complications. The presence of placentomegaly, cardiomegaly, or non-immune hydrops fetalis is indicative of a poor outcome [15]. Fetal MRI may provide additional anatomical information [16, 17]. Antenatal pickup was extremely poor in our patients (2/10).

Mode of delivery: LSCS is advised to mothers whose fetuses harbour large SCTs (>10 cm diameter) and highly vascular tumours; this is done after fetal lung maturity has been attained [5, 18]. Others recommend LSCS for all SCTs larger than 5cm in order to prevent the risk of rupture and bleeding [19]. Despite 5 tumours being >10 cm in dimension, only 2 mothers underwent LSCS in our series. This could have led to the high incidence of tumour rupture (2/10). As 8/10 SCTs were not antenatally diagnosed, the issue of elective LSCS to prevent rupture of large tumours, did not arise. It is incredulous that 2 of the mothers delivered their babies having large SCTs through normal vaginal route at home and unsupervised. Nevertheless, similar reports of large SCTs being born normally at home are found from other third world countries as well [20]. Tumour rupture has been shown to be associated with increased perinatal mortality [15].

Antenatal intervention: In contrast to neonatal SCT, prenatally diagnosed SCT remains at a high risk of perinatal complications and death [21]. The presence of placentomegaly/ hydrops in such a fetus heralds impending fetal demise. High-output cardiac failure resulting from arteriovenous shunting through the tumour is postulated to be the cause for fetal death [22]. Unless the shunt is reversed, it is difficult to save the fetus. Thus an urgent surgical intervention in the form of tumour debulking, either as fetal surgery or postnatally after urgent Caesarean section, is indispensable. Definitive SCT resection can be carried out in the neonatal period after stabilization. In 1997, Adzick et al reported the first successful fetal resection of SCT in a 26 week old fetus with a large SCT, polyhydromnios and impending hydrops; thus reversing the pathophysiology of vascular steal and preventing hydrops [23]. Surprisingly, Graf et al found that 5 preterm SCT debulking specimens all showed grade 3 immature teratomas and 2 also had nests of malignant foci. On comparing the HPE with definitive resection specimens, they actually found tumour maturation in all 5 cases with disappearance of malignant foci, presence of mature histology and absence of any SCT tissues (only fibrosis). Whether these favourable changes were induced by the preterm debulking or they represent natural maturation of SCT during gestation, is food for thought [24]. Successful antenatal decompression of a cystic Type IV SCT using an amniotic catheter, to assuage bilateral hydronephrosis and renal damage, has also been described [25]. Other antenatal interventions include aspiration of the cyst and amnioreduction which are usually done to ease maternal discomfort from gross distension and also to avoid preterm labour [21]. Kum et al have described the following prerequisites before prenatal intervention for SCT in preterm pregnancy [18]:

1. Accurate prenatal diagnosis and well defined natural history to allow the confident diagnosis of a correctable lesion that will otherwise prevent fetal survival.
2. Absence of other life threatening or debilitating anomalies.
3. Ability to perform the procedure without increased risk to the mother’s life or her future fertility.

While we are still struggling to achieve accurate prenatal diagnosis, fetal intervention appears far-fetched in our current scenario. 

Postnatal Surgical Excision: Excision within the 1st week of life should be the aim whenever possible. It is important to confirm the availability of cross-matched blood in the operating room before starting the surgery since even benign SCTs can cause significant blood loss and exsanguination on the table [26]. Most on table deaths during SCT resection are because of cardiac arrest resulting from electrolyte imbalance (hyperkalaemia), and massive bleeding during surgery. Hence, a careful anaesthetic management is essential [27]. Complete excision of the tumour with coccygectomy is of paramount importance in preventing recurrence. The recurrence rates reported without removal of the coccyx are as high as 37% [9]. The most common approach used is sacral (44.4%) [11] as was done in all our cases. However, in large tumors and especially in Type III and IV SCTs, one should not hesitate to take the sacro-abdominal approach (22.6%) [11]. 

Complications: The complications seen in our patients are comparable to that reported in literature with wound infection being the commonest due to proximity of the surgical site to the anus [5, 6]. Neuropathic bladder has also been reported [6, 28]. The temporary diarrhoea (9%) usually settles by itself in majority of the cases as was seen in 2 of our patients [5]. However, bowel incontinence and constipation have been described when these patients are followed up to adulthood [29]. The neurological problems are more likely to occur when intra-pelvic tumour has been removed surgically. In a 30-year follow-up study on 25 patients operated for SCT, Bittmann et al found that poor cosmetic results in the buttock region was the most common long-term complication [30]. Other authors have reported lack of cosmesis in 29 – 40 % patients [4, 31]. This can lead to distortion of body image, particularly in teenagers, and can even cause psychological disturbances and depression. Providing good cosmesis may be challenging in the neonatal period due to lack of local tissues, large tumours, thinned/ stretched out muscles, problems with skin flaps and high chances of postoperative wound infection. However, once the tumour has been tackled and there is no recurrence for few years, plastic surgical reconstruction must be considered in these children including the use of silicone implants for flat gluteal regions. 

Malignancy: The chances of malignancy increase when the tumor size is >10 cm [1, 32], in Altman Type III and IV due to delay in diagnosis, with the presence of solid areas [5, 15] and when presentation is beyond the 2nd month of life [1]. Incidence of malignancy in SCTs larger than 10 cm is 17% [1].

Survival: Excellent survival rates of more than 95%, similar to our series, have been reported in literature for neonatal SCTs [23]. Mortality rate for SCTs larger than 10cm in dimension is reported as 18% [1]. 

Recurrence: It is important to remember that even completely excised mature neonatal SCT has a startling potential to recur (11-22%) either as a benign or malignant tumour during the first 3 years of life [11, 32]. Close follow-up every 3-6 months with physical examination including rectal examination, serum alpha-fetoprotein, and diagnostic imaging, is advisable for at least 3 years [32]. Raised AFP levels may be the first indicator for recurrence even without any obvious local recurrence [32]. However, the follow-up must be continued into adult life as Lahdene et al have reported a 6.7% recurrence, 21 – 43 years after resection of neonatal SCT [33]. The reasons for recurrence after a mature teratoma has been excised could be numerous. Large tumours may possibly have imperceptible tiny foci of malignant endodermal sinus cells which are mistakenly reported on HPE as mature [32]. Incomplete resection of tumour, intra-operative tumour spillage and omission of coccygectomy also increases the occurrence of malignant changes in the recurrent tumour [5, 9, 34]. Another possibility is malignant degeneration occurring in a mature teratoma which is known to happen with increasing age. Spillage of cyst fluid however does not predispose to recurrence [34].

Unfortunately, despite meticulous counselling and advice, only 40% of our patients came for regular follow-up. There was no response to our reminder letters either. In all probability, the children who are LTFU are well. Hence uneducated, poor, parents, who are living hand-to-mouth, feel it is an unnecessary effort to bring the child for a medical check-up. Nevertheless, since we have not seen these patients, we cannot comment on their follow-up.

## CONCLUSION

The importance of early diagnosis and treatment of any sacrococcygeal mass, no matter how small or apparently insignificant it may appear cannot be overemphasized. There is a potential to lead a normal life without disability after neonatal excision of SCT. However long term follow-up is advised as recurrences have occurred even in adulthood.

## Footnotes

**Source of Support:** Nil

**Conflict of Interest:** One of the authors’ belongs to editorial board; however, the manuscript was independently handled by other editors. The authors are not involved in any decision making regarding this manuscript.
